# Ultrasound Versus Magnetic Resonance Imaging as First-Line Imaging Strategies for Rotator Cuff Pathologies: A Comprehensive Analysis of Clinical Practices, Economic Efficiency, and Future Perspectives

**DOI:** 10.7759/cureus.59231

**Published:** 2024-04-28

**Authors:** Yvana Toh

**Affiliations:** 1 Surgery, Surgical Treatment and Rehabilitation Service (STARS), Brisbane, AUS

**Keywords:** healthcare burden, chronic shoulder pain, shoulder, mri, us, rotator cuff, magnetic resonance imaging, ultrasound

## Abstract

Rotator cuff injuries are a prevalent cause of atraumatic chronic shoulder pain, imposing a significant healthcare burden. This article reviews the clinical presentation, diagnostic imaging modalities, practice variations, and economic efficiency considerations in the evaluation of rotator cuff pathologies.

Ultrasound (US) and magnetic resonance imaging (MRI) are the primary imaging methods for diagnosing rotator cuff injuries. US provides real-time visualization but has limited tissue penetration, while MRI offers detailed anatomical information but is not a dynamic process. Studies show that MRI is superior to US with higher sensitivity and specificity. MRI is the gold standard, particularly for surgical planning, but US remains relevant when MRI is not feasible. Both require standardized protocols for evaluating tear dimensions and muscle atrophy. With the operator-dependent nature of US, MRI offers a more comprehensive assessment of rotator cuff tears and predictive insights for clinical outcomes.

Practice variations exist in the management of rotator cuff pathologies, with some countries favoring US as the primary imaging modality and others relying more on MRI. These variations are influenced by factors like resource availability and healthcare system nuances. In Australia, current guidelines lean toward conservative management, potentially leading to delayed diagnoses and increased costs. The United States often favors MRI, while Canada advocates for US as the initial choice.

Economic considerations play a significant role in selecting imaging modalities. While US is cost-effective, it may necessitate subsequent MRI examinations, contributing to inefficiencies in the diagnostic process. Studies suggest that a combined approach of US and MRI is less efficient and cost-effective than MRI alone. However, the use of both modalities rather than MRI alone is common in clinical practice, adding to healthcare expenses.

In conclusion, the choice of imaging modality for rotator cuff pathologies should consider factors such as diagnostic efficacy, cost-effectiveness, and resource availability. Radiologists play a pivotal role in guiding this selection and ensuring comprehensive evaluations. Future considerations should include the revision of management guidelines and the potential inclusion of shoulder pathologies in healthcare coverage to optimize patient care and healthcare expenditure.

## Introduction and background

Rotator cuff injuries are the most common cause of atraumatic chronic shoulder pain [1]. Rotator cuff-related shoulder pain is the most common shoulder condition presented in general practitioner settings, contributing significantly to the healthcare burden [2]. Streamlining the diagnosis of rotator cuff tears is crucial for guiding treatment approaches aimed at pain relief, reducing extended disability, and minimizing overall healthcare costs. According to the current Royal Australian College of General Practitioners (RACGP) guidelines, first-line management of suspected rotator cuff injury is through clinical exam and early referral to a physiotherapist [1]. The benefit of imaging and referral to an orthopedic specialist is of debatable benefit [3]. The most sensitive first-line imaging modality to identify rotator cuff pathology includes ultrasound (US) and magnetic resonance imaging (MRI) [4]. This article aims to utilize existing literature to outline a pragmatic method for imaging rotator cuff pathologies.

Learning objectives

The objectives of this article are (1) providing a comprehensive understanding of two common imaging modalities, such as US and MRI, including their strengths and limitations, and familiarizing standardized protocols for MRI and US examinations in the context of rotator cuff injuries; (2) emphasizing the importance of standardization in diagnosis and its impact on clinical outcomes; (3) understanding practice variations in different healthcare systems and recognizing how healthcare system factors and reimbursement policies can influence imaging choices; (4) assessing the cost-effectiveness of imaging approaches for rotator cuff injuries by weighing the balance between cost efficiency and diagnostic accuracy; and (5) recognizing the role of radiologists in promoting effective treatment strategies and recommending appropriate imaging studies for rotator cuff injuries.

## Review

Clinical presentation and anatomy of rotator cuff

**Figure 1:**
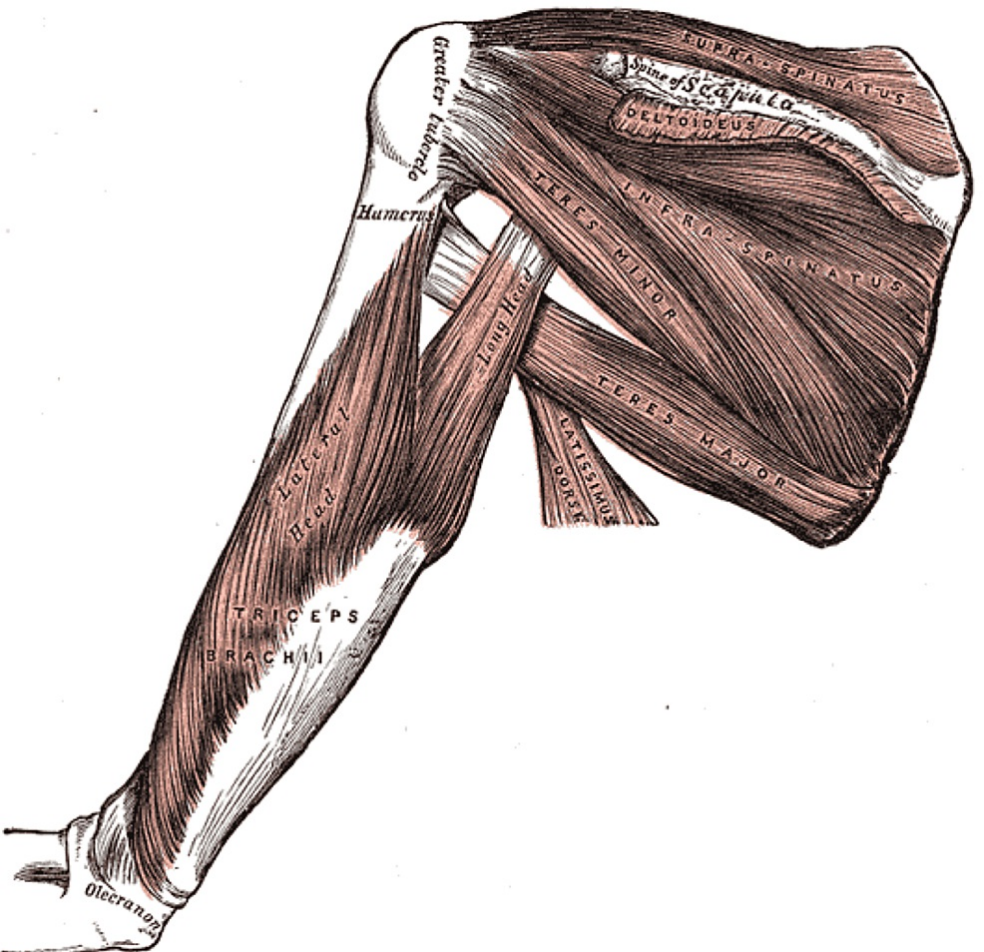
Shoulder muscle anatomy (posterior view) Image source: Ref. [7].

**Figure 2:**
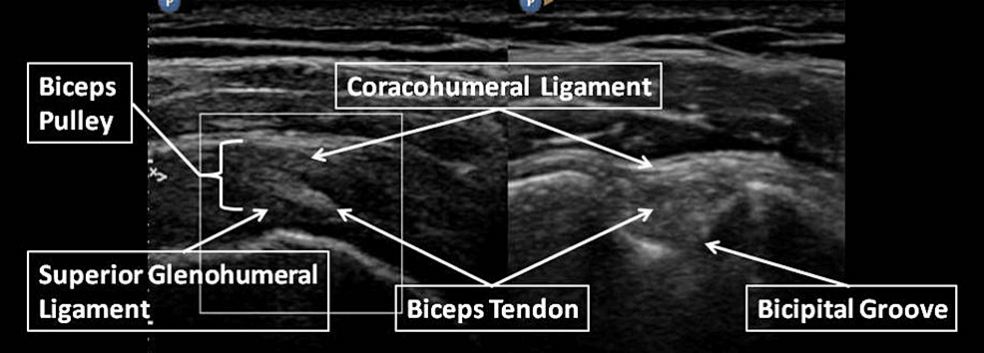
Anatomy of shoulder joint seen on ultrasound Image source: Ref. [8].

The shoulder is a complex ball and socket joint largely stabilized by the rotator cuff allowing multidirectional mobility. The shoulder joint encompasses the humeral head and the shallow glenoid fossa of the scapula. The rotator cuff, a confluence of four muscles (including the supraspinatus, infraspinatus, teres minor, and subscapularis), collectively fosters stability and facilitates controlled rotation of the humeral head within the glenoid cavity [4]. These muscles are intrinsically linked as a continuous structure encapsulating multiple facets of the humeral head, which transmit forces and movement from the muscles to the bone [5]. The biomechanics of the shoulder joint are a manifestation of its complex interactions. The humeral head's convexity engages the glenoid's concavity, effectuating its range of motion. The glenoid labrum, a fibrocartilaginous rim encircling the glenoid cavity, enhances joint stability and depth, augmenting the humeral head's congruency within the socket. The deltoid muscle envelopes the joint, accentuating its dynamic capabilities, while collaborating with the rotator cuff for harmonious movement (Figures 1, 2) [6].

The shoulder joint's intricate architecture renders it susceptible to an array of injuries, with rotator cuff tears being the commonest pathology. Taking the above points into consideration, insertional rotator cuff lesions are complex pathologies involving multiple regions, either involving the anterior portion of the supraspinatus tendon along with the anterior subscapularis tendon or alternatively encompassing the posterior portion of the supraspinatus tendon along with the anterior part of the infraspinatus tendon. The most common cause of rotator cuff tear is due to the biomechanical phenomenon of impingement [4]. Repetitive activity causes recurrent microtrauma, muscular fiber disruption, and subsequent regeneration. Impingement predominantly manifests in the anterosuperior sector of the rotator cuff, which is situated between the greater tuberosity of the humerus and the acromion during shoulder movements involving flexion, abduction, and external rotation [6]. There are also rarer impingement syndromes, such as the one occurring between the posterosuperior aspect of the greater tuberosity and the posterior glenoid (internal impingement or posterosuperior impingement) or within the distal subscapularis tendon, positioned between the coracoid process and the lesser tuberosity of the humerus (known as subcoracoid impingement) [6].

**Figure 3:**
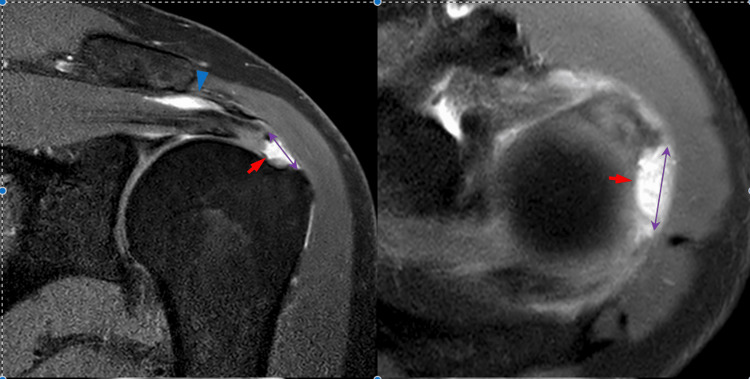
Full-thickness rotator cuff supraspinatus tear on MRI Left: Coronal view of shoulder joint on MRI. The blue arrowhead indicates a full-thickness/transmural tear of the distal supraspinatus tendon (red arrow) with increased fluid in the subacromial/subdeltoid bursa. Right: Shoulder joint MRI axial view T1. The red arrowhead shows mild long-head biceps tendinosis. The purple arrow indicates the measurement of the mediolateral and anteroposterior diameter of the supraspinatus muscle. Image source: Ref. [10].

**Figure 4:**
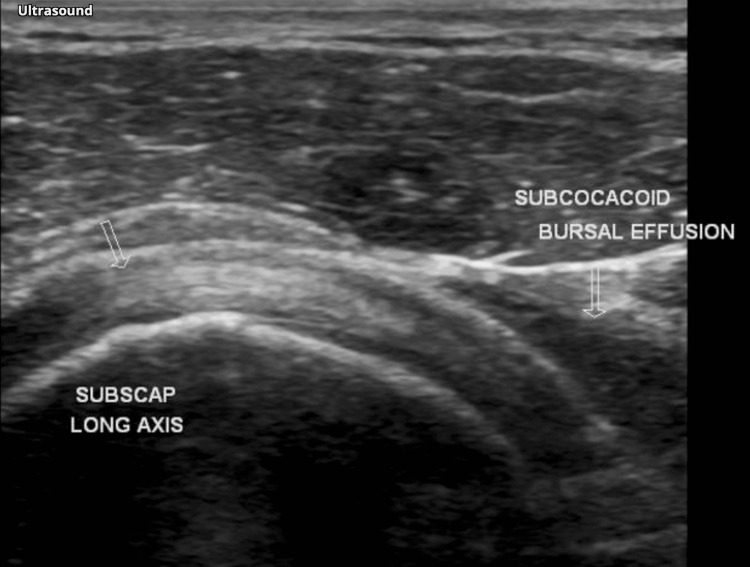
Transverse ultrasound of the shoulder joint revealed complete tears in both the subscapularis and supraspinatus tendons as indicated by an arrowhead Image source: Ref. [11].

Conceptualizing these impingement patterns as a framework during the interpretation of medical imaging aids in achieving precise characterization of rotator cuff injuries. For instance, when reviewing MRI results, the presence of tendon degeneration within the anterior supraspinatus tendon extending to the superior edge of the subscapularis tendon, along with the presence of bursal fluid and a subacromial spur, supports a diagnosis of anterosuperior rotator cuff impingement, even though the MRI cannot directly visualize the impingement itself. In contrast, US can directly visualize this impingement by imaging the subacromial bursa that overlays the anterosuperior cuff during shoulder abduction [9]. However, it was unable to provide an accurate assessment of the magnitude of the supraspinatus tendon tear or offer a precise depiction of the subacromial spur's involvement in impingement [4]. This highlights impingement as a dynamic process, and a radiologist should aim to incorporate this perspective into their imaging reports, regardless of the imaging modality used. Restricting the radiological characterization and management of rotator cuff tears to a single contributing tendon is a misdirection. Instead, the focus should be directed toward the intricacies of rotator cuff morphology and the dynamic complexity of its biomechanical design (Figures 3, 4).

Imaging modalities

In the assessment of rotator cuff pathologies, two primary imaging modalities such as US and MRI are commonly used [4]. These modalities, both recommended as the initial diagnostic tools, bestow distinct and complementary imaging virtues, thereby enhancing the diagnostic continuum. While ultrasound excels in real-time visualization, its proficiency is constrained by tissue penetration limitations. Conversely, MRI offers meticulous anatomical detail but may not capture dynamic processes with equivalent precision. Regardless of the chosen imaging modality, both MRI and US examinations should adhere to a standardized protocol. This standardized MRI protocol is typically conducted without contrast at magnetic field strengths of 1.5 and 3 Tesla and ought to encompass a minimum of three anatomical planes (coronal oblique, sagittal oblique, and axial). It should also incorporate water-sensitive sequences with fluid sensitivity and fat suppression, along with at least one T1-weighted sequence to facilitate precise assessment and differentiation between bone and soft tissue windows (Table 1) [4,12].

**Table 1:** Sample non-contrast shoulder 3T MRI protocol FOV: Field of view; FS: Fat-suppressed; PD: Proton density; TE: Echo time; TR: Repetition time; 3T: 3-Tesla; MRI: Magnetic resonance imaging.

Sequence	TR (ms)	TE (ms)	Slice thickness (mm)	FOV (mm)	Matrix
Axial PD FS	3000	30	3.0	130	320 × 288
Coronal oblique T1	1000	10	3.0	130	288 × 256
Coronal oblique T2 FS	3500	70	3.0	130	320 × 288
Sagittal oblique T1	600	10	3.0	130	288 × 256
Sagittal oblique T2 FS	3500	70	3.0	130	320 × 288

US is a technically demanding and operator-dependent imaging modality. Its inherent dynamism underscores the importance of an adept operator for the comprehensive evaluation of pathological anomalies and the attainment of precise diagnoses. Typically employing a high-frequency probe (7-12 MHz) to achieve requisite anatomical resolution, the examination begins with the patient seated, their arm supinated and maintained at a neutral position, with the elbow flexed at 90 degrees. Evaluating each aspect of the rotator cuff requires positioning the patient optimally, often adopting the impingement position, to enhance visualization of the intricate structures comprising the rotator cuff (Table 2) [4,13].

**Table 2:** Sample of shoulder ultrasound protocol

Structure	View	Position	Pathologies detected
Extraarticular long-head biceps tendon	Longitudinal and transverse	Neutral with supinated palm resting on the knee	Tendinosis, tear, tenosynovitis, dislocation/subluxation
Subscapularis	Longitudinal and transverse	External rotation	Tendinosis, tear, fluid in subscapularis bursa
Supraspinatus tendon	Longitudinal and transverse	Modified Crass position	Tendinosis, tear
Infraspinatus tendon	Longitudinal and transverse	Hand on contralateral shoulder	Tendinosis, tear
Subacromial bursa	Coronal plane at the lateral margin of the acromion	Neutral with arm abducted to shoulder level	Fluid in bursa and impingement with abduction
Posterior glenohumeral joint	Axial plane	Neutral	Joint effusion, hyperemia, osteoarthritis
Posterior rotator cuff muscle bellies	Sagittal plane	Neutral	Muscle atrophy of infraspinatus or teres minor
Spinoglenoid notch	Axial plane	Neutral	Paralabral cyst or mass
Acromioclavicular joint	Axial plane	Neutral	Osteoarthritis, capsular hypertrophy, hyperemia

Liu et al. conducted a comprehensive meta-analysis comprising 144 studies, which revealed that unenhanced 3-Tesla MRI offers superior diagnostic value compared to high-frequency ultrasound (≥7.5 MHz), followed by unenhanced 1.5-Tesla MRI and low-frequency ultrasound (<7.5 MHz). Notably, in the detection of any rotator cuff tear, MRI exhibited superior performance compared to US, with higher sensitivity (0.84 vs. 0.81), specificity (0.86 vs. 0.82), and superiority index (0.98 vs. 0.22), respectively. When focusing on full-thickness tears, MRI demonstrated higher sensitivity and superiority index relative to US (0.91 vs. 0.87 and 0.67 vs. 0.28, respectively), while maintaining a similar level of specificity (0.88 vs. 0.88, respectively) [14].

Standardization of rotator cuff tear definitions is essential for achieving comprehensive and quantifiable assessments. Tears should be measured in anterior-posterior and transverse dimensions to ensure a thorough evaluation. Massive rotator cuff tears denote full-thickness involvement encompassing at least two-thirds of the rotator cuff attachment on the greater tuberosity along with tendon retraction reaching the glenoid rim. This is frequently associated with macroinstability and glenohumeral osteoarthritis [15,16]. In cases of full-thickness rotator cuff tears, the degree of muscle atrophy should be systematically graded using established systems like the Goutallier classification, which ranges from grade 0 representing normal muscle to grade 4 indicating more than 50% fatty muscle atrophy, correlating with poorer post-repair outcomes [17]. For partial-thickness tears, quantification should involve determining the percentage of tendon depth affected, with tears exceeding 50% of the tendon depth typically classified as high grade. However, the severity assessment may also consider factors such as tear location and extent. In cases of larger tears, it is imperative to measure the maximal transverse retraction of the proximal end of the torn tendons as clinical outcomes tend to worsen as tendon retraction occurs along with increased risk of muscle atrophy [15]. When assessing the scope of rotator cuff tears, MRI surpasses US in its ability to provide a comprehensive evaluation, offering detailed insights and categorization of tear extent, and predictive information regarding clinical outcomes. MRI affords a more intricate visualization of anatomical structures, thereby enhancing surgical planning and approach considerations, including the assessment of bone loss that can necessitate alterations in surgical techniques and postoperative rehabilitation protocols. While studies have demonstrated the high diagnostic value of high-frequency US, approaching comparability with MRI [14], US cannot match the precision of MRI in delineating individual muscle bulk loss. However, it can effectively identify and report on fatty replacement and overall atrophy of rotator cuff muscles, particularly when incorporating extended field of view (FOV) techniques [18].

Taking the aforementioned factors into consideration, musculoskeletal imaging practitioners are encouraged to harness all accessible resources to formulate an imaging report that enhances the diagnostic evaluation and treatment strategy. The comprehensive report should encompass essential patient demographics, a meticulous examination of trauma history and presenting symptoms, potential influences from occupational and recreational activities, and any pertinent prior imaging records. Particularly in healthcare settings lacking integrated electronic medical record systems and imaging archives, diligent efforts should be exerted to secure prior imaging studies and incorporate them into the interpretation of the current imaging assessment, thereby facilitating the creation of a holistic evaluation and judiciously guiding subsequent management decisions.

Factors influencing imaging modalities

When devising the imaging strategy for patients presenting with suspected rotator cuff pathologies, a comprehensive evaluation of various factors becomes imperative. These factors encompass the patient's individual profile, the accessibility of resources, cost implications, and the diagnostic efficacy of each modality, all of which collectively contribute to the formulation of an optimal imaging protocol.

Both MRI and US prove valuable in the evaluation of patients with suspected rotator cuff tears. Utilizing US as the primary imaging modality following initial radiography offers several advantages, including its generally lower cost compared to MRI, the ability to correlate imaging findings with point tenderness, dynamic maneuver visualization, contralateral extremity comparison, and the feasibility of real-time guided interventions [19]. Furthermore, when considering aspects such as patient comfort, pain perception, and the duration of the imaging modality, a substantial preference for US over MRI emerges. In fact, patient satisfaction rates reveal that 78% favored US, while only 6% expressed a preference for MRI [20]. With its notable sensitivity and specificity, particularly for full-thickness tears, US stands as a robust and cost-effective first-line imaging choice. Furthermore, in healthcare systems characterized by limited per capita resources and lower prioritization of non-emergent musculoskeletal examinations, extended wait times for shoulder MRI studies are common [21]. Consequently, US emerges as a more accessible and economically prudent option for initial imaging assessment in cases of suspected rotator cuff pathology.

MRI presents numerous advantages over US in the evaluation of rotator cuff pathologies. MRI demonstrates higher interobserver reliability in assessing tear size and tendon retraction compared to US, making it the preferred choice for surgical planning [22]. Its diagnostic efficacy, particularly when utilizing standardized protocols well-known to technologists, radiologists, and interpreters, surpasses that of US. Nonetheless, obtaining high-quality musculoskeletal US necessitates specialized skills and optimal performance by credentialed musculoskeletal US technologists, with interpretation by experienced radiologists [23]. This requirement may constrain the accessibility and overall diagnostic utility of US. Liu et al. conducted a comprehensive meta-analysis of 144 studies, underscoring the superior diagnostic value of unenhanced 3-Tesla MRI over high-frequency ultrasound (≥7.5 MHz). MRI consistently demonstrated superior performance with heightened sensitivity and specificity in detecting rotator cuff tears [14]. However, it is worth noting that MRI has recognized contraindications and may pose challenges in specific patient populations, such as those with claustrophobia or severe obesity. Despite potential mobility limitations, shoulder US remains virtually unrestricted in terms of contraindications [9]. Tenbrunsel et al. asserted that MRI remains the gold standard for rotator cuff tear imaging and is a superior predictor of clinical outcomes. While US plays a role in reducing healthcare costs, its precision is somewhat compromised due to inherent limitations in comparison to MRI [18].

While both MRI and US provide valuable insights into rotator cuff pathologies, MRI emerges as the gold standard due to higher reliability, superior diagnostic value, and effectiveness in surgical planning. Nevertheless, US continues to hold significance, particularly in scenarios where MRI is contraindicated or accessibility is restricted, thus contributing to cost-effective healthcare strategies. In this evolving landscape, radiologists play a pivotal role in spearheading the development of impactful and efficient imaging algorithms, thereby shaping the future of imaging guidelines and best practices.

Practice variations

Current management guidelines in Australia, as outlined by the RACGP for rotator cuff pathologies, primarily recommend a thorough clinical examination focused on the shoulder and early referral of patients to physiotherapy and non-steroidal anti-inflammatories (NSAID). While imaging may be considered an indicative measure, the guidelines do not place a strong emphasis on obtaining precise diagnostic investigations to delineate the pathology [3]. It is noteworthy that these guidelines were last updated in 2012, and numerous scientific studies conducted since then have provided compelling evidence regarding the efficacy and significance of diagnostic imaging. This evidence has further advanced our understanding of the treatment process and enhanced management outcomes. Additionally, this approach to management incurs higher costs compared to utilizing an appropriate imaging modality with potential input from orthopedic specialists. A study conducted by Friedman et al. revealed that 56.9% of patients initially treated conservatively subsequently required a more invasive form of treatment [24].

Naunton et al. conducted an extensive five-year study that examined the prevalence, management strategies, and economic impact of rotator cuff pain managed by general practitioners (GPs) in South Australia. Their findings revealed that rotator cuff pain represents the most frequently encountered shoulder condition, accounting for 5.12 cases per 1,000 medical encounters, with an estimated national incidence of 732,000 cases. Predominantly, the management of rotator cuff pain among GPs involved medications (54.7%), followed by steroid injections (19.5%) and non-steroidal anti-inflammatory drugs (NSAIDs) (19.1%). Notably, imaging was requested for 43.4% of all rotator cuff presentations, US (41.2%) and X-ray (11.6%). None of the patients underwent an MRI. The unwarranted use of imaging in rotator cuff-related pain contributes to the rising healthcare costs associated with diagnostic procedures and poses potential harm to patients. The collective imaging expenses for shoulder ultrasound and X-ray procedures were estimated at $60 million and $23 million, respectively [1].

An existing obstacle to adopting MRI as the primary imaging modality in Australia lies in the absence of financial support from Medicare, the publicly funded universal healthcare insurance system in the country. At present, only three anatomical regions, namely the head, cervical spine, and knee, are covered under the Medicare Benefits Scheme [25]. It is hoped that, in the future, the inclusion of shoulder pathologies in this scheme will be considered, given the compelling evidence supporting its cost-effectiveness in the initial management of rotator cuff pathologies.

In the United States, MRI is frequently employed as the primary imaging choice for evaluating rotator cuff pathologies. Orthopedic surgeons initiate the diagnostic process with unenhanced MRI, particularly among patients under 40 years of age. Additionally, MRI is often favored for the initial postoperative assessment of shoulder conditions, with only a minority of patients being referred to US. One potential obstacle to the broader integration of shoulder US in the United States is its reliance on routine hours, necessitating the presence of subspecialty technologists and radiologists. In contrast, MRI scheduling offers greater flexibility, including evening and weekend hours [26].

In Canada, US is frequently employed as the primary imaging modality for initial evaluation. Choosing Wisely Canada campaign advocates for the use of first-line US in assessing rotator cuff and bursal pathologies, reserving MRI, or magnetic resonance arthrography (MRA) for cases where labral pathology is suspected [27]. Nevertheless, it is common practice for orthopedic surgeons to request a preoperative MRI examination to assess the degree of retraction and muscle atrophy, especially if the initial US report indicates a full-thickness tear and the patient is a surgical candidate. Furthermore, unenhanced MRI remains the preferred initial test for evaluating the integrity of rotator cuff repair [5].

Economic efficiency

Although US is often favored for its cost efficiency, it raises questions regarding its comprehensive diagnostic efficacy as a significant portion of patients still require subsequent MRI examinations for a thorough diagnostic workup, precise pathology characterization, and potential surgical planning.

Gyftopoulos et al. constructed a model considering the cost of diagnostic imaging and treatment to predict both cost and quality-adjusted life years in the management of full-thickness supraspinatus tears. The study revealed that US as a standalone approach was the most cost-effective, but MRI alone is the most efficacious strategy. The combination of initial US followed by MRI, while more costly than US alone, proved to be less effective than MRI alone, thus not recommended as an optimal approach [21]. Regrettably, the prevailing practice involves the simultaneous utilization of both US and MRI in the assessment of most patients with rotator cuff pathology, a practice that not only lacks efficiency but also adds to the burgeoning healthcare burden.

Garwood et al. assessed the efficacy of MRI in comparison to X-rays. It was found that 97.8% of MRI studies were classified as useful, primarily exhibiting a high level of utility. In contrast, X-rays were observed to have the lowest utility or were deemed non-useful in 85.5% of cases. Nonetheless, it is important to note that a direct and meaningful comparison between US and MRI in this study was not feasible due to the substantial disparity in the availability of different imaging modalities within the initial dataset, which included 159 X-ray, 137 MRI, two CT, and four ultrasound examinations [28].

## Conclusions

In summary, optimizing the imaging approach for rotator cuff pathologies requires a careful evaluation of various factors, including cost-effectiveness, resource availability, and diagnostic efficacy. While MRI stands as the preferred choice for its diagnostic value, US is beneficial, offering real-time visualization and is cost-effective. Radiologists hold a critical position in facilitating the choice of optimal imaging modalities tailored to precise clinical contexts, thereby enhancing treatment efficacy and advising on suitable imaging protocols for rotator cuff injuries. Their expertise contributes significantly to formulating imaging reports that enhance the diagnostic evaluation and facilitate the development of effective treatment strategies.
